# *dTrmt10A* impacts Hsp70 chaperone m^6^A levels and the stress response in the *Drosophila* brain

**DOI:** 10.1038/s41598-023-50272-4

**Published:** 2023-12-28

**Authors:** Alexandra E. Perlegos, Xiuming Quan, Kirby M. Donnelly, Hui Shen, Emily J. Shields, Heidi Elashal, Kathy Fange Liu, Nancy M. Bonini

**Affiliations:** 1 Neuroscience Graduate Group, Perelman School of Medicine, University of Pennsylvania, Philadelphia, PA 19104 USA; 2 Department of Biology, University of Pennsylvania, Philadelphia, PA 19104 USA; 3 School of Life Science and Technology, China Pharmaceutical University, Nanjing, 210009 Jiangsu China; 4 Epigenetics Institute, Perelman School of Medicine, University of Pennsylvania, Philadelphia, PA USA; 5 Department of Cell and Developmental Biology, Perelman School of Medicine, University of Pennsylvania, Philadelphia, PA USA; 6 Department of Biochemistry and Biophysics, Perelman School of Medicine, University of Pennsylvania, Philadelphia, PA 19104 USA; 7 Graduate Group in Biochemistry and Molecular Biophysics, Perelman School of Medicine, University of Pennsylvania, Philadelphia, PA 19104 USA

**Keywords:** Epigenetics, RNA sequencing, Genetics, Neuroscience, Molecular biology, RNA metabolism, Transcriptomics

## Abstract

Chronic cellular stress has a profound impact on the brain, leading to degeneration and accelerated aging. Recent work has revealed the vital role of RNA modifications, and the proteins responsible for regulating them, in the stress response. In our study, we defined the role of *CG14618*/*dTrmt10A*, the *Drosophila* counterpart of human *TRMT10A* a *N*^*1*^-methylguanosine methyltransferase, on m^6^A regulation and heat stress resilience in the *Drosophila* brain. By m^6^A-IP RNA sequencing on *Drosophila* head tissue, we demonstrated that manipulating *dTrmt10A* levels indirectly regulates m^6^A levels on polyA + RNA. *dTrmt10A* exerted its influence on m^6^A levels on transcripts enriched for neuronal signaling and heat stress pathways, similar to the m^6^A methyltransferase *Mettl3*. Intriguingly, its impact primarily targeted 3' UTR m^6^A, setting it apart from the majority of *Drosophila* m^6^A-modified transcripts which display 5' UTR enrichment. Upregulation of *dTrmt10A* led to increased resilience to acute heat stress, decreased m^6^A modification on heat shock chaperones, and coincided with decreased decay of chaperone transcripts and increased translation of chaperone proteins. Overall, these findings establish a potential mechanism by which *dTrmt10A* regulates the acute brain stress response through m^6^A modification.

## Introduction

RNA modifications are emerging as a critical layer of gene expression that impact various biological functions in the brain, such as learning and memory, neurogenesis, and developmental transitions^1–7^. Mutations in methylation complex proteins and dysregulation in RNA modification can lead to severe developmental defects, compromised cellular stress responses, and tumorigenesis^8–10^. TRMT10A is an S-adenosylmethionine-dependent methyltransferase that installs the RNA modification *N*^*1*^-methylguanosine (m^1^G) on tRNAs at the ninth position; it is the only known methyltransferase that installs m^1^G on human tRNA^11,12^. tRNAs are among the most heavily modified classes of RNA and these modifications are critical for the stability and translation functionality of tRNAs^11,12^. Mutations of *TRMT10A* in humans have been linked to primary microcephaly, mild intellectual disability, young onset diabetes, and apoptosis in pancreatic cells^13–17^. However, the mechanisms by which genetic mutations in *TRMT10A* cause these phenotypes are not well understood.

N^6^-methyladenosine (m^6^A) is the most abundant internal modification of eukaryotic mRNA^1,18,19^. It is deposited co-transcriptionally by a methyltransferase, METTL3, with the help of additional structural proteins. m^6^A levels are dynamically regulated by writer, reader, and eraser proteins^8,19^. The m^6^A modification of mRNA provides a critical layer of regulation in neuronal responses to cellular stress and disease^20–22^. Interaction of m^6^A transcripts with reader proteins can dictate downstream processing, including splicing, decay, and translation^23–26^. TRMT10A has been shown to interact with the m^6^A RNA demethylase FTO and promote its demethylase activity in HEK293T cells^27^. Loss of TRMT10A increases m^6^A methylation on FTO RNA targets in cultured cells^27^. m^6^A levels are enriched in brain tissue of human, mice, and *Drosophila*, and mark transcripts involved in synaptic growth, axon guidance and signaling^22,28–30^. m^6^A reader proteins, or YTH domain-containing proteins, are known for their ability to promote translation and decay of m^6^A-modified RNAs^7,31–33^. One of their characterized functions is the ability to regulate cortical neurogenesis through precise decay of neuronal m^6^A transcripts during developmental transitions in mouse and human cells^30,33^.

During cellular stress, the role of m^6^A modifications becomes amplified with increased methylation dynamically regulating RNAs for rapid degradation and/or selective translation^20,28,34–37^. During UV DNA damage stress, m^6^A methylation functions in recruitment of DNA damage machinery^38^, and during heat stress transcripts become marked for decay and translation in in vitro human cell culture models^39–41^. Neurons are critically vulnerable to prolonged stress and degenerative disease. Heat shock protein chaperones (HSPs) are known to protect neuronal cells against protein misfolding, accumulation and aggregation, but HSPs are dysregulated during aging and disease^42^.

*Drosophila* is a powerful genetic model system that has pioneered numerous fundamental discoveries in brain development and disease. Homologous m^6^A methyltransferase complex components are enriched in the *Drosophila* central nervous system (CNS), making the fly an ideal system for understanding m^6^A modification in brain functions^19^. In *Drosophila*, m^6^A is implicated in female specific alternative splicing of the master sex determination regulatory gene *Sex-lethal* (*Sxl*)^23,43,44^. Additionally, the *Drosophila* cytoplasmic m^6^A reader protein Ythdf regulates axonal overgrowth through interactions with Fmr1, the fly homolog of Fragile X mental retardation protein, and together they inhibit translation of m^6^A modified targets of Fmr1^45^. Furthermore, there is no identified homologous m^6^A demethylase in *Drosophila*.

Here, we investigate the fly homolog of *TRMT10A*, *CG14618* (which we now refer to as *dTrmt10A*)*,* to uncover its regulation of m^6^A levels and the stress response in the *Drosophila* brain. The role of *TRMT10A*/*dTrmt10A* and its effects on m^6^A levels have not been studied in vivo. Our m^6^A-IP sequencing results indicate that *dTrmt10A* impacts m^6^A on signaling transcripts, and alters m^6^A on key heat stress chaperone transcripts^3,46^. We find that upregulation of *dTrmt10A* acts similarly to loss of m^6^A methyltransferase *Mettl3* in the *Drosophila* brain, and highlights m^6^A peaks in the 3’UTR of transcripts. These findings indicate that *dTrmt10A* modulates m^6^A in *Drosophila* and plays a critical role in regulating the stress response of the brain.

## Results

### m^6^A is enriched in the brain and ***dTrmt10A*** knockdown increases m^6^A levels on Hsp70 transcripts.

**Figure 1:**
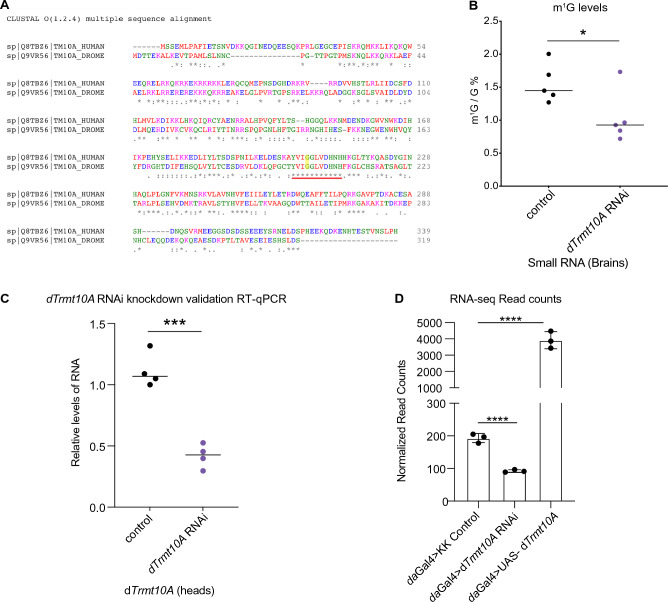
Impact of the *Drosophila* homolog of TRMT10A on m^1^G and m^6^A levels. (**A**) Alignment of Human TRMT10A and *Drosophila* dTrmt10A/CG14618 by Clustal Omega multiple sequence comparison. Asterisk * indicates fully conserved residues, a colon indicates conservation of strongly similar properties, and period indicates conservation of weakly similar properties. Protein alignment shows overall 36% conservation between *Drosophila* and human protein. Red line indicates conserved m^1^G catalytic domain, and G206R mutation highlighted in yellow indicates catalytic mutant. (**B**) LC–MS/MS analysis of m^1^G/G% levels from brain small RNA in basal conditions, in control vs *dTrmt10A* RNAi, n = 5 biological replicates, 30 brains per replicate. Data are presented as mean, t-test *p < 0.05. (**C**) RNA levels of *dTrmt10A* were assessed by RT-qPCR. *da*Gal4 > UAS-KK control (Vienna 60100); *da*Gal4 > UAS-*dTrmt10A* RNAi (Vienna 100720); n = 3 biological replicates, 15 brains per replicate. Data are presented as mean, t-test, ***p < 0.001. (**D**) RNA levels of *dTrmt10A* determined from RNA-sequencing *Drosophila* head tissue, normalized read counts and statistical analysis done by Deseq2. See Supplementary Data 1 for details of fly genotypes in all figures.

The *Drosophila* gene *CG14618* is the predicted homolog of the human *TRMT10A* methyltransferase. Using Clustal Omega, human TRMT10A and *Drosophila* CG14618 share 36.8% amino acid sequence identity, with 100% conservation in the m^1^G catalytic domain "YVIGGLVDHNH" (Fig. 1A). We refer to *CG14618* as *Drosophila Trmt10A* (*dTrmt10A*) from here on. Knockdown of *dTrmt10A *in vivo produced viable offspring. Using liquid chromatography-tandem mass spectrometry (LC–MS/MS), we confirmed significant m^1^G level reduction in *dTrmt10A* RNAi brains (small RNA fraction < 200 nucleotides), compared to control brains (Fig. 1B). *dTrmt10A* mRNA levels in head tissue were significantly reduced by RT-qPCR and RNA-seq analysis (Fig. 1C,D and Supplementary Data 1).

**Figure 2:**
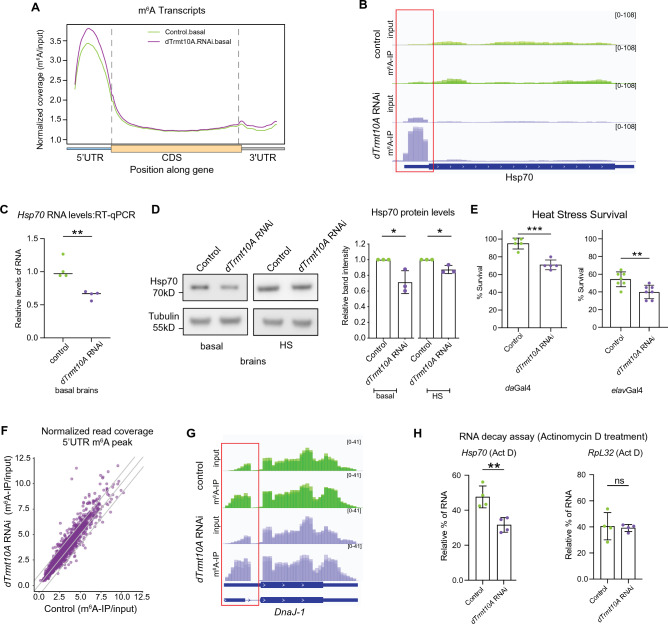
*dTrmt10A* knockdown increases m^6^A on the 5′UTR of *Hsp70* transcripts. (**A**) Normalized read coverage plot of m^6^A-IP/input on polyA + transcripts in the 5′UTR, CDS, and 3′UTR of m^6^A genes. m^6^A-IP sequencing in basal conditions from fly heads (*da*Gal4 > KK control vs *da*Gal4 > *dTrmt10A* RNAi). (**B**) IGV genome browser tracks of m^6^A-IP and Input reads for *Hsp70* genes in basal conditions, from control and *dTrmt10A* RNAi. Red box highlights increase of 5’UTR m^6^A with *dTrmt10A* RNAi. (**C**) RT-qPCR shows decreased levels of Hsp70 RNA in basal conditions from KK control and *dTrmt10A* RNAi brains. t-test, **p < 0.01. (**D**) *dTrmt10A* RNAi decreased protein levels of Hsp70 in basal and HS conditions. t-test, *p < 0.05. (**E**) *da*Gal4 > KK control vs *da*Gal4 > *dTrmt10A* RNAi and *elav*Gal4 > KK control vs *elav*Gal4 > *dTrmt10A* RNAi increased heat stress sensitivity of the animals. Animals heat stressed for 1 h at 38.5 °C and allowed to recover for 24 h. t-test, ***p < 0.001. **p < 0.01. (**F**) Scatterplot of normalized read coverage of m^6^A-IP/input for 5′UTR of m^6^A transcripts in control versus *dTrmt10A* RNAi conditions. *dTrmt10A* RNAi shows a shift towards increased m^6^A levels in 5’UTR. (**G**) Genome browser tracks of m^6^A-IP and Input reads for *DnaJ-1* in basal conditions, from control and *dTrmt10A* RNAi. Red box highlights increased 5’UTR m^6^A upon *dTrmt10A* upregulation. RADAR differential peak analysis in Supplemental Data 2. (**H**) RNA decay assay from Control and *dTrmt10A* RNAi fly brains, dissected and incubated in Schneider’s *Drosophila* Medium plus 169.5ug/ml of actinomycin D for 4 h. RNA was extracted and used for RT-qPCR to determine relative RNA levels of *RpL32*, and *Hsp70*. Data are presented as mean ± SD of n = 4 biological replicates, 12 brains per replicate. **p < 0.01. Student’s two-tailed t-test. ns = not significant.

Given that TRMT10A interacts with FTO in mammalian cells^27^, we conducted m^6^A-IP sequencing analysis of polyA + RNA from *Drosophila* heads expressing *dTrmt10A* RNAi. IP sequencing confirmed that m^6^A is enriched in the 5'UTR of transcripts in *Drosophila* heads, consistent with our and others previous work^3,46^. Enrichment of m^6^A in the 5'UTR is unique to *Drosophila*, as mammalian m^6^A is primarily located in the 3'UTR^28^. Global metagene analysis highlighted that *dTrmt10A* knockdown increased m^6^A levels in the 5'UTR of transcripts, and unexpectedly also showed increased m^6^A in the 3'UTR of a subset of transcripts (Fig. 2A).

To determine which transcripts showed significant differential methylation between control and *dTrmt10A* knockdown conditions, we utilized RADAR analysis with a beta-threshold of 0.5. Using these parameters, only transcripts from a single gene showed a significant increase in m^6^A. These transcripts encode the heat shock chaperone protein Hsp70 (Fig. 2B). We were particularly intrigued by this finding as there are normally low levels of m^6^A in the 5'UTR on *Hsp70* transcripts at baseline, and m^6^A signal is lost upon acute heat stress in the *Drosophila* brain^46^. Previously, we found that knockdown of m^6^A methyltransferase *Mettl3* showed increased RNA and protein levels of Hsp70 at baseline along with increased heat stress resilience^46^. We asked if an opposite effect occurred with knockdown of *dTrmt10A*. In *dTrmt10A* RNAi brains, RNA and protein levels of Hsp70 were reduced (Fig. 2C,D). Additionally, *dTrmt10A* RNAi animals exhibited increased stress sensitivity (Fig. 2E). These data indicate that *dTrmt10A* RNAi exerts contrasting effects on Hsp70 RNA, protein, and m^6^A levels compared to *Mettl3* RNAi.

To visualize global changes in m^6^A, regardless of peak calling threshold, we generated a scatterplot of m^6^A-IP reads divided by input read coverage in the 5'UTR for *Mettl3*-dependent m^6^A transcripts. Upon *dTrmt10A* knockdown, we observed a global shift towards increased levels of m^6^A in the 5'UTR of transcripts (Fig. 2F), similar to the global metagene analysis (Fig. 2A). We observed a slight increase of 5’UTR m^6^A on other chaperones such as Heat shock chaperone 40 (*DnaJ-1*) (Fig. 2G), but no changes comparable to the increase on *Hsp70 5’UTR*. These findings highlight the regulation of *Hsp70* transcripts by the m^6^A machinery, and indicate it as key target of dTrmt10A.

To analyze the impact of m^6^A on RNA levels with *dTrmt10A* manipulation, we employed an ex-vivo brain RNA decay assay. Reduced m^6^A by *Mettl3* RNAi lead to higher steady state levels and decreased rate of decay of m^6^A transcripts^46^. We examined the relative levels of RNA remaining in *dTrmt10A* RNAi brain samples after 4 h, in the presence of actinomycin D. *dTrmt10A* RNAi brains (with increased m^6^A) had increased decay of *Hsp70* RNA transcript levels compared to control brains, and no significant difference of *RpL32,* which is a control non-m^6^A modified transcript (Fig. 2H). These data suggest that d*Trmt10A* influences the stability of Hsp70 transcripts and overall protein and RNA levels, likely by modulating their m^6^A levels.

### Upregulation of *dTrmt10A* decreases m^6^A levels and enhances the stress response of the brain.

**Figure 3:**
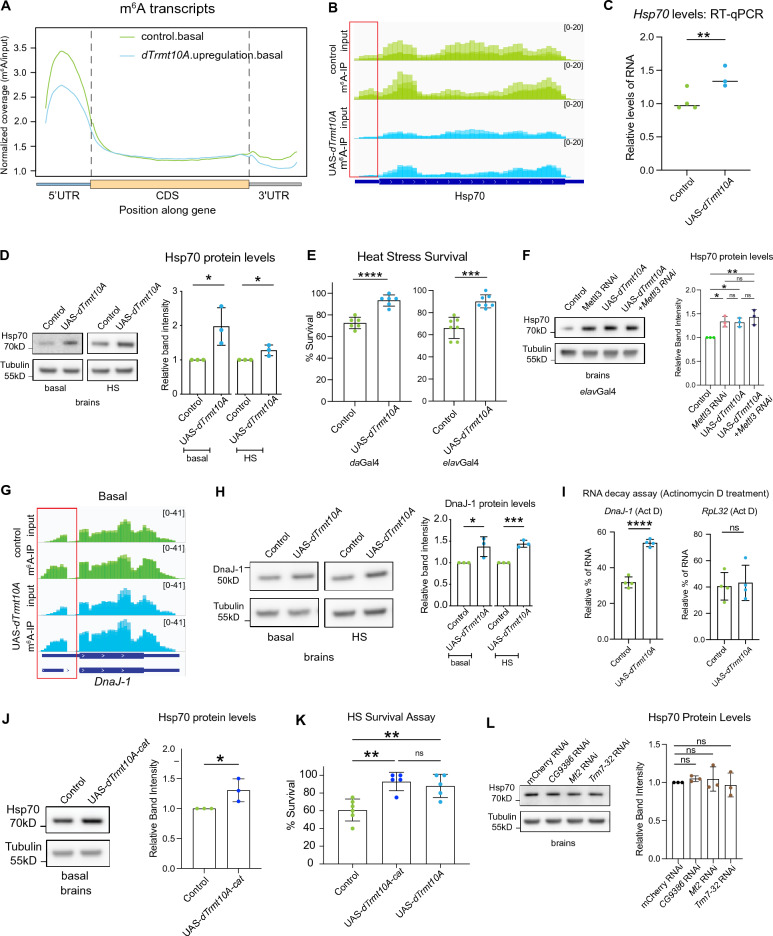
*dTrmt10A* upregulation confers heat stress resilience. (**A**) Normalized read coverage plot of m^6^A-IP/input on polyA + transcripts in the 5’UTR, CDS, and 3’UTR of m^6^A modified transcripts. m^6^A-IP sequencing in basal conditions from control fly (*da*Gal4 > KK control) versus *dTrmt10A* upregulation (*da*Gal4 > UAS-*dTrmt10A*) heads. (**B**) IGV genome browser tracks of m^6^A-IP enrichment versus Input reads for Hsp70 in basal conditions, from control (*da*Gal4 > KK control) and *dTrmt10A* upregulation (*da*Gal4 > UAS-*dTrmt10A*). Red box highlights loss of 5’UTR m^6^A with *dTrmt10A* upregulation. (**C**) RT-qPCR of Hsp70 RNA levels in basal conditions from control (*da*Gal4 > KK control) and *dTrmt10A* upregulation (*da*Gal4 > UAS-*dTrmt10A*) brains. t-test, **p < 0.01. (**D**) Upregulation of *dTrmt10A* increased protein levels of Hsp70 in basal and heat stress conditions. *da*Gal4 > KK control vs *da*Gal4 > UAS-*dTrmt10A*, *elav*Gal4 > KK control vs *elav*Gal4 > UAS-*dTrmt10A* t-test, *p < 0.05. (**E**) Upregulation of *dTrmt10A* (*da*Gal4 > Control vs. UAS-*dTrmt10A* or *elav*Gal4 > Control vs UAS-*dTrmt10A*) increased animal heat stress survival. t-test, ****p < 0.0001, ***p < 0.001. (F) Hsp70 protein levels from brains. *elav*Gal4;mCherry RNAi > UAS-mcd8GFP vs *elav*Gal4;*Mettl3* RNAi > UAS-mcd8-GFP vs *elav*Gal4;mCherry RNAi > UAS-*dTrmt10A* vs ElavGal4;*Mettl3* RNAi > UAS-*dTrmt10A,* n = 3 biological replicates, *p < 0.05, **p < 0.01 One-way ANOVA. (**G**) Genome browser tracks of m^6^A-IP and Input reads for *DnaJ-1* in basal conditions, from control and UAS-*dTrmt10A*. Red box highlights loss of 5’UTR m^6^A with *dTrmt10A* upregulation. (**H**) Upregulation of *dTrmt10A* increased protein levels of DnaJ-1 in basal and HS conditions. t-test, *p < 0.05, ***p < 0.001. RADAR differential peak analysis in Supplemental Data 2. (**I**) RNA decay assay from Control, and UAS-*dTrmt10A*, in the presence of actinomycin D, to determine relative RNA levels of *RpL32* and *DnaJ-1*. Data are presented as mean ± SD of n = 4 biological replicates, 12 brains per replicate. ****p < 0.0001. Student’s two-tailed t-test. ns = not significant. (**J**) UAS-*dTrmt10A-cat* increased protein levels of Hsp70 in basal brain conditions. *da*Gal4 > KK control vs *da*Gal4 > UAS-*dTrmt10A-cat*, t-test, *p < 0.05. (**K**) *da*GAL4 > *U*AS-*dTrmt10A-cat* mutant increased heat stress resilience of the animals. Animals heat stressed for 1 h at 38.5 °C and allowed to recover for 24 h. One-way ANOVA, **p < 0.01. (**L**) Hsp70 protein levels in basal brains with knockdown of additional tRNA methyltransferases. *da*Gal4 > mCherry RNAi, *da*Gal4 > *CG9386* RNAi, *da*Gal4 > *Mt2* RNAi, *da*Gal4 > *Trm7-32* RNAi, One-way ANOVA, ns = not significant.

Studies investigating the role of *TRMT10A* in cultured cells have focused on mutations or gene downregulation^17,27^. To determine whether upregulation of *dTrmt10A* could also regulate m^6^A levels, we utilized a FLAG-HA C-terminal tagged ORF clone of *dTrmt10A* to generate animals that upregulate *dTrmt10A* (Supplementary Fig. 1A). Upregulation of *dTrmt10A* showed no global effect on m^1^G levels, likely due to the saturation of m^1^G levels on tRNAs (Supplementary Fig. 1B). m^6^A-IP sequencing analysis conducted on *Drosophila* heads with *dTrmt10A* upregulation revealed a striking reduction in m^6^A levels (Fig. 3A). A closer look at heat stress chaperones revealed 5’UTR m^6^A normally present at baseline on *Hsp70* transcripts was lost upon *dTrmt10A* upregulation, conferring the opposite effect from *dTrmt10A* RNAi (Fig. 3B). Additionally, Hsp70 RNA and protein levels were elevated in UAS-*dTrmt10A* brains (Fig. 3C,D), and animals were more resilient to heat stress (Fig. 3E). Loss of *Mettl3* also leads to elevated Hsp70 transcript and protein levels. To provide support that the change in Hsp70 with *dTrmt10A* upregulation was due to effects on m^6^A, vs other possibilities, we simultaneously reduced *Mettl3* and upregulated *dTrmt10A* in neurons. Co-expression of *Mettl3* RNAi and UAS-*dTrmt10A* in the brain showed a similar increase in Hsp70 protein levels compared to expression of either transgene alone (Fig. 3F). These data suggest that *dTrmt10A* is acting through the m^6^A/*Mettl3* pathway to modulate Hsp70 levels.

Similar to *Mettl3* RNAi brains, *dTrmt10A* upregulation significantly reduced 5’UTR m^6^A on *DnaJ-1(*Hsp40 chaperone), and UAS-*dTrmt10A* brains had increased protein levels of DnaJ-1 (Fig. 3G,H). RNA decay analysis indicated upregulation of *dTrmt10A* led to higher stability of *DnaJ-1* RNA while there was no significant difference observed in the RNA levels of the unmodified *RpL32* transcript (Fig. 3I). Overall, *dTrmt10A* upregulation showed similar effects on heat shock chaperone levels and heat shock resilience as observed with *Mettl3* RNAi^46^. Elevated levels of stress chaperones at baseline in the brain potentially precondition the brain to acute heat stress. Taken together, these data highlight that upregulation of *dTrmt10A* reduces m^6^A levels and confers heat stress resilience.

Because dTrmt10A is a tRNA methyltransferase, we performed additional experiments to confirm that its role to modulate Hsp70 and the stress response is due to an impact on m^6^A, and not its role as a tRNA methyltransferase. We generated animals that expressed *dTrmt10A* with a known mutation in the conserved m^1^G catalytic domain of the methyltransferase activity that abolishes tRNA methyltransferase activity in vitro^14^ (G206R; see Fig. 1A). We assessed the impact of this mutation using sensitive readouts of *dTrmt10A* function: the levels of Hsp70 and animal stress sensitivity. Baseline levels of Hsp70 were increased in the brains of the animals expressing the catalytic mutant (UAS-*dTrmt10a*-*cat*), and these animals had increased stress resilience comparable to wild type (Fig. 3J,K). These data indicate that the phenotypic effects of *dTrmt10A* on Hsp70 and stress resilience are not dependent on the m^1^G catalytic activity. We also examined three additional tRNA methyltransferases for effects on Hsp70 levels. RNAi of these additional tRNA methyltransferases (*CG9386* RNAi, *Mt2* RNAi, *Trm7-32* RNAi) had no effect on basal Hsp70 levels in the brain (Fig. 3L). Together these data indicate that the effects of *dTrmt10A* on Hsp70 levels and stress resilience are independent of its catalytic activity as a tRNA methyltransferase, and are not a general property of compromised tRNA methyltransferase activity.

### Upregulation of *dTrmt10A* mimics loss of methyltransferase *Mettl3*, and globally decreases m^6^A on neurogenesis and signaling transcripts.

**Figure 4:**
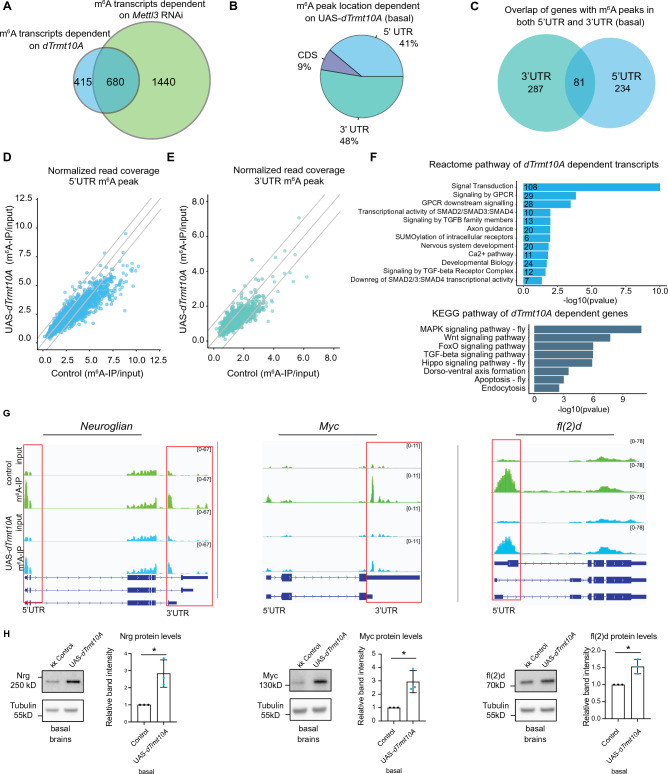
*dTrmt10A* upregulation decreases m^6^A on neurogenesis and signaling pathway transcripts. (**A**) Overlap of *dTrmt10A*-dependent m^6^A from the m^6^A-IP sequencing experiment, and *Mettl3*-dependent m^6^A transcripts (from^46^). We focused on the 680 transcripts that are overlapping as *dTrmt10A*-dependent targets. (**B**) Percent location of *dTrmt10A*-dependent m^6^A at baseline indicates an enrichment for 3’UTR m^6^A regulation. (**C**) Overlap of *dTrmt10A* m^6^A targets with 5’UTR peaks and those with 3’UTR peaks; some transcripts have m^6^A in both locations. List of genes given in Supplemental Data 2. (**D**–**E**) Scatterplot of normalized read coverage of m^6^A-IP/input for 5’UTR (left) or 3’UTR (right) of m^6^A transcripts in control versus UAS-*dTrmt10A* conditions. *dTrmt10A* upregulation shows a shift towards decreased m^6^A levels. (**F**) Pathway analysis and Kegg term^49,50^ analysis of all *dTrmt10A*-dependent m^6^A transcripts shows enrichment for signaling and neuronal pathways. (**G**) IGV genome browser tracks of m^6^A-IP enrichment versus Input reads for *Nrg*, *Myc,* and *fl(2)d* in basal conditions, from control and UAS-*dTrmt10A*. Red box highlights loss of 5’UTR or 3’UTR m^6^A with *dTrmt10A* upregulation. (**H**) Nrg, Myc, fl(2)d protein levels from control or UAS-*dTrmt10A* brains dissected in basal conditions. Quantification of 3-biological replicate immunoblots, 15 brains per replicate, showing increased expression of protein in (*da*Gal4 > UAS-*dTrmt10A)* fly brain. Data are presented as mean ± SD, t-test *p < 0.05.

A total of 675 transcripts showed differential m^6^A methylation upon *dTrmt10A* upregulation, and only a few of these transcripts were HS chaperones (Fig. 4A). To investigate *dTrmt10A* further, we compared the m^6^A sites from *dTrmt10A* upregulation m^6^A-IP to the defined *Mettl3*-dependent methyltransferase m^6^A sites and transcripts^46^. 62% of the transcripts from genes with *Mettl3*-dependent m^6^A were also regulated by *dTrmt10A* (Fig. 4A). We focused on these transcripts, as m^6^A is *Mettl3*-dependent. Closer analysis of these methylated transcripts revealed 41% of *dTrmt10A*-dependent m^6^A sites were in the 5'UTR, while 48% were in the 3'UTR (Fig. 4B), with a small number (81 genes) showing both 5'UTR and 3'UTR-dependent m^6^A changes (Fig. 4C). Modulation of m^6^A on the 3’UTR of transcripts was surprising due to the enrichment of m^6^A on the 5’UTR in *Drosophila*^3,46–48^. This finding suggests that *dTrmt10A* may have a specialized role to regulate 3'UTR m^6^A. When we examined all *Mettl3*-dependent m^6^A transcripts with peaks in either the 5’UTR (Fig. 4D) or 3’UTR (Fig. 4E) regions; there was a similar shift towards decreased m^6^A-IP/Input enrichment upon *dTrmt10A* upregulation for both regions of transcripts. Reactome and KEGG^49,50^ pathway analyses showed that these transcripts are enriched for dynamic signaling pathways and axon guidance pathways (Fig. 4F and Supplementary Data 3). Similar GO terms were enriched regardless of 5’UTR or 3’UTR modification site.

We examined select targets with available antibodies (*Nrg*, *Myc,* and *fl(2)d*) to determine whether *dTrmt10A*-dependent m^6^A modification of transcripts impacted protein levels. Upregulation of *dTrmt10A* showed decreased m^6^A on these transcripts, and we observed increased protein levels in *Drosophila* brain tissue (Fig. 4G,H). These findings support previous work in mammalian cells that observe changes in Myc levels due to m^6^A-TRMT10A interaction^27^. We extended these data to global protein synthesis using a puromycin assay to assess nascent protein synthesis. These data showed that upregulation of *dTrmt10A* was associated with increased puromycin incorporation, thus increased global nascent protein translation in the brain (Supplementary Fig. 1D). We observed the opposite response in puromycin levels upon *dTrmt10A* RNAi (Supplementary Fig. 1D). The catalytic mutant animals still showed increased global protein synthesis in the brain by puromycin assay (Supplementary Fig. 1E), confirming that puromycin changes are not due to m^1^G activity of dTrmt10A. The additional tRNA methyltransferases, which did not impact Hsp70 protein levels, also did not affect global puromycin incorporation (Supplementary Fig. 1F). Therefore, *dTrmt10A* modulation of m^6^A transcripts likely leads to the influence on overall protein levels in the brain.

Many chromatin regulatory RNAs are m^6^A modified, and m^6^A methyltransferase complex components are known to bind chromatin and regulate transcription elements and histone modifications^51,52^. We therefore considered whether dTrmt10A may also be associated with chromatin. *dTrmt10A* was overexpressed in the *Drosophila* salivary glands and dTrmt10A protein localization was examined on the large polytene chromosomes by immunostaining to the HA tag. dTrmt10A showed robust localization to the nucleolus as anticipated for its role in tRNA modification; however, dTrmt10A also showed binding to band locations on the polytene chromosomes (Supplementary Fig. 2A). Chromatin immunoprecipitation followed by western immunoblot validated dTrmt10A association with chromatin (Supplementary Fig. 2B) and chromatin binding was not dependent on RNA (Supplementary Fig. 2C). These data indicate that dTrmt10A is positioned for an additional layer to m^6^A-modified gene regulation on chromatin, similar to Mettl3^51,53^.

### With stress, *dTrmt10A* primarily impacts 3’UTR m^6^A sites

**Figure 5:**
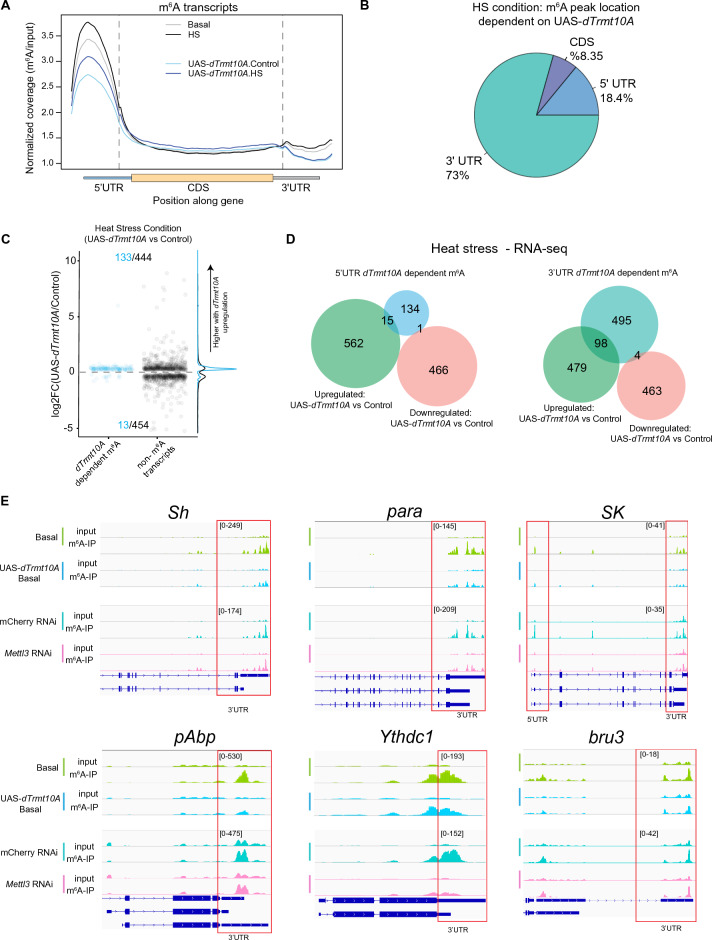
*dTrmt10A* upregulation shows more 3’UTR m^6^A peak changes with heat stress (HS). (**A**) Normalized read coverage plot of m^6^A-IP/input on polyA + transcripts in the 5’UTR, CDS, and 3’UTR of m^6^A modified transcripts. m^6^A-IP sequencing in basal and HS conditions from control (*da*Gal4 > KK control) versus *dTrmt10A* upregulation (*da*Gal4 > UAS-*dTrmt10A*) heads. (**B**) Location of *dTrmt10A*-dependent m^6^A indicates enrichment for 3’UTR m^6^A regulation upon HS. (**C**) Plot of significantly differentially expressed genes in *da*Gal4 > KK control vs > UAS-*dTrmt10A* heads in HS condition. Positive logFC indicates an increase in transcript levels in *dTrmt10A* upregulation heads. m^6^A genes (blue), or all other genes (black). (**D**) Venn diagram of 5’UTR or 3’UTR *dTrmt10A* m^6^A marked transcripts with genes that are differentially expressed in control vs UAS-*dTrmt10A* in HS conditions. (**E**) IGV genome browser tracks of m^6^A-IP enrichment versus Input reads for transcripts in basal conditions, from KK control and UAS-*dTrmt10A*. Bottom four lanes depict IGV tracks from mCherry RNAi control and *Mettl3* RNAi in basal conditions. These peaks show dependence on *Mettl3* and UAS-*dTrmt10A* by RADAR analysis. Red box highlights 3’UTR m^6^A. Values denote data range on IGV browser for either UAS-*dTrmt10A* or *Mettl3*.RNAi sequencing experiments.

Based on the heat stress resilience of upregulated *dTrmt10A*, we looked more closely at the regulation of m^6^A during heat stress with overexpression of *dTrmt10A*. We performed acute heat shock (HS) experiments then analyzed m^6^A-IP from *Drosophila* head polyA + RNA. These data showed that *dTrmt10A* upregulation reduced overall m^6^A levels in both basal and HS conditions (Fig. 5A). Interestingly, *dTrmt10A* upregulation led to modification of mostly 3'UTR m^6^A sites during heat stress, with 73% of *dTrmt10A*-dependent sites located in this region of transcripts upon HS (Fig. 5B).

Upon heat stress and *dTrmt10A* upregulation, there was a shift towards increased expression of m^6^A marked transcripts (Fig. 5C, blue vs black). Furthermore, the majority of these differentially expressed transcripts exhibited *dTrmt10A*-dependent m^6^A modifications on their 3' UTR (Fig. 5D). Notably, there was no trend in the levels of non-m^6^A modified transcripts, with equal numbers of transcripts being up and downregulated between control and *dTrmt10A* upregulation (Fig. 5C). Among the m^6^A transcripts, we identified critical genes involved in signaling processes and neurogenesis, such as *pAbp*, *SK*, *para*, *Sh, bru3* and even the nuclear m^6^A reader protein *Ythdc1* (Fig. 5E and Supplementary Fig. 3A). These findings highlight the essential role of *dTrmt10A* in m^6^A modification during stress, particularly in modifying transcripts that are integral to neuronal signaling processes.

Compared to other tissues, many genes in the *Drosophila* nervous system have substantially extended 3’UTRs and alternative polyadenylation^54,55^. We found that 25% of *Drosophila* CNS transcripts with extended 3’UTRs also have m^6^A modification in their 3’UTR (Supplementary Fig. 3B). Furthermore, this subset of m^6^A transcripts are enriched for cognition, learning, and memory pathways, indicating this subclass of 3’UTR m^6^A modified transcripts are alternatively polyadenylated and involved in learning and memory. We also examined the overall length of 3’UTRs of m^6^A modified transcripts and found that the average length of their 3’UTR was longer compared to all 3’UTR weighted lengths from wildtype *Drosophila* CNS^55^ (Supplementary Fig. 3C).

### Proteins that interact with dTrmt10A

**Figure 6:**
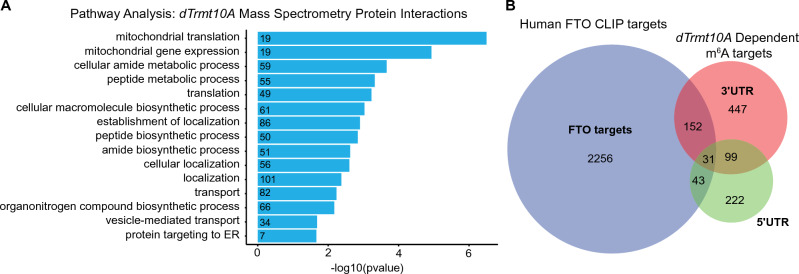
dTrmt10A co-IP interactions from head lysates. (**A**) Affinity purification of HA–tagged dTrmt10A followed by protein mass spectrometry to identify dTrmt10A-associated proteins. GO term analysis of proteins shown to have a positive interaction with dTrmt10A. Genes above 1.3 LogFC selected as positive interaction. −log10(pval) enrichment of genes and number of genes in each category is shown. See Supplementary Data 4 for full protein list and GO terms with *p*-values. (**B**) Human FTO CLIP targets (Ontiveros, R Jordan et al. 2020^27^), overlapped with m^6^A gene targets modified in the 5′UTR or 3′UTR m^6^A by upregulation of *dTrmt10A*. Mass spectrometry proteins and GO analysis genes listed in Supplemental Data 4.

In mammalian cells, TRMT10A regulates m^6^A by interacting with the m^6^A demethylase FTO. Although there are no known homologs in *Drosophila* to the demethylases FTO or ALKBH5, we considered that a fly demethylase, should it exist^24,27,56^, may interact with dTrmt10A. To define interacting proteins, we expressed UAS-*dTrmt10A-HA* and immunoprecipitated dTrmt10A from *Drosophila* head lysates using the HA tag. Mass spectrometry was used to evaluate enriched protein interactions of dTrmt10A-HA compared to IgG control (Supplementary Data 4). This analysis identified 6 high confidence interacting proteins (Pp1-87B, CG18680, CG9953, hrm, Dhx15), with no apparent homologs to FTO or ALKBH5. When we analyzed all interactions (low, medium, and high), there was enrichment for proteins involved in RNA metabolism and deadenylation, including DCP1, Atx2, twin, and Rga, as well as proteins involved in translation regulation, such as eIF3a (Fig. 6A). These proteins may interact with and regulate downstream m^6^A-modified RNA processing through interaction with dTrmt10A. We also investigated whether dTrmt10A binds or alters the levels of known m^6^A regulators such as Mettl3 or m^6^A reader proteins Ythdc1 or Fmr1, but found no interaction or altered protein levels (Supplementary Fig. 4), indicating that dTrmt10A does not interact with these known m^6^A binding proteins to mediate its effect on m^6^A.

Both mammalian and *Drosophila* m^6^A-modified transcripts are enriched for similar biological pathways^22,30,57^, highlighting the importance of m^6^A in signaling and stress. Although a candidate demethylase is not identified in *Drosophila*, we used FTO CLIP targets from mammalian cells^27^ to define the homologous *Drosophila* genes. We compared these FTO CLIP targets to *dTrmt10A*-dependent m^6^A transcripts and found that ~ 23% of *dTrmt10A*-dependent m^6^A transcripts overlap with FTO targets (Fig. 6B). This overlap is similar to the extent of overlap of transcript targets between TRMT10A and FTO in mammalian cells (35%^27^). Notably, 3’UTR m^6^A transcripts showed a greater overlap with FTO targets (Fig. 6B). These data suggest that the interaction of dTrmt10A toward 3’UTR m^6^A RNA binding proteins and/or a putative demethylase may fine-tune aspects of RNA processing.

## Discussion

**Figure 7:**
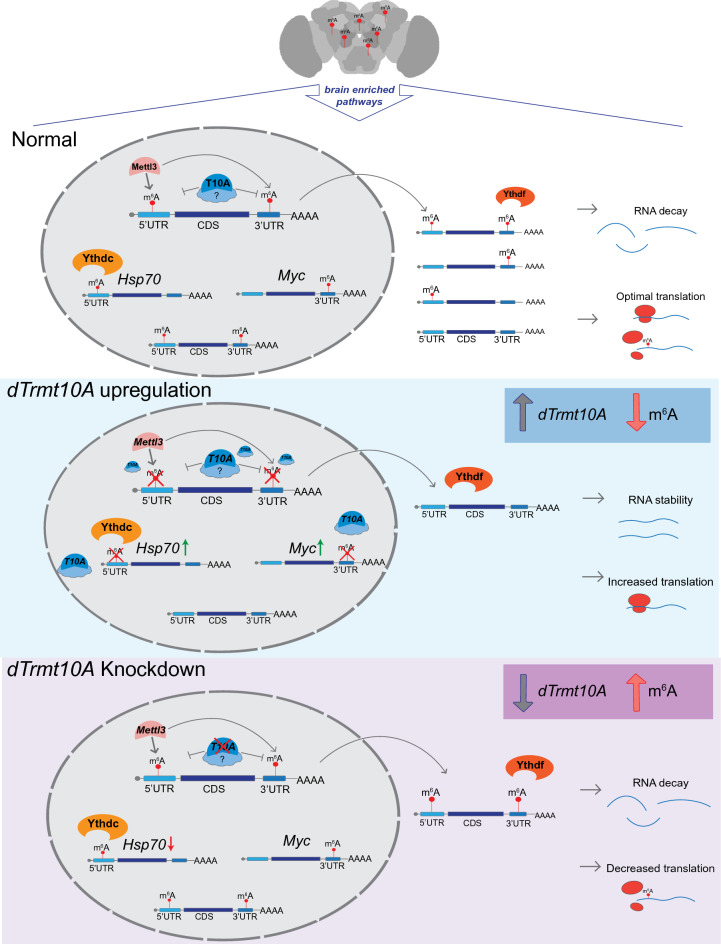
Model of *dTrmt10A* regulation in the brain. m^6^A is highly enriched in the *Drosophila* brain, and marks transcripts enriched in dynamic signaling and neuronal growth pathways. *dTrmt10A* upregulation reduces m^6^A levels in the 5’UTR and 3’UTR of transcripts. *Myc* and *Hsp70* are example genes that show increased protein levels with *dTrmt10A* upregulation. *dTrmt10A* upregulation leads to increased RNA and protein levels of these transcripts. Conversely, *dTrmt10A* knockdown shows a dramatic increase in 5’UTR m^6^A, with significance for *Hsp70*. *dTrmt10A* upregulation modulates heat stress chaperones of *Drosophila* such that animals are more stress resilient.

This study offers valuable insights into the in vivo role of *dTrmt10A* on m^6^A modification within the *Drosophila* brain, and its impact on stress resilience and gene regulation. Of note, *dTrmt10A* modulates m^6^A peaks both on the 5’UTR of transcripts (where most m^6^A peaks are in *Drosophila* transcripts), but also on the 3’UTR of *Drosophila* genes. Upregulation of *dTrmt10A* caused a global decrease in m^6^A levels on transcripts and increased the animal’s resilience to heat stress, with increases in overall protein and RNA levels of key players like Hsp70 (see Figs. 3 and 4). These findings highlight that *Drosophila* transcripts are modulated by m^6^A in the 3’UTR in addition to 5’UTR, confirming that m^6^A modification plays a crucial role in dampening the biological response of the brain to acute stress, and that *dTrmt10A* acts as an additional regulator of m^6^A modification of transcripts in the brain. Moreover, modulation of *dTrmt10A* showed the ability to modulate m^6^A peak levels in transcripts, despite not being a canonical component of the methyltransferase complex (Fig. 7). dTrmt10A with a mutation in the m^1^G catalytic domain of the tRNA methyltransferase functioned similarly in our assays as the wild-type protein, and additional *Drosophila* tRNA methyltransferases failed to show these phenotypes, confirming that the function of dTrmt10A as a tRNA methyltransferase is not influencing Hsp70 protein levels and protein translation. There are many known mutations in human TRMT10A that impact brain development^13–15^, and these data now indicate that the function of TRMT10A/dTrmt10A on m^6^A levels may also play a role in these diseases.

### m^6^A modification in brain homeostasis

Many biological functions in the brain are regulated by RNA modification, including axon growth, embryonic stem cell fate, learning and memory, and neurogenesis^30,58^. Furthermore, mutations in RNA modification proteins have been linked to compromised cellular stress responses, tumorigenesis, and severe developmental defects^9,10,20,33,38^. Our findings contribute to the current knowledge of RNA modifications regulating the neuronal response to cellular stress, and the regulation of m^6^A overall in the brain. *dTrmt10A* regulated m^6^A levels on signaling pathway transcripts, such as MAPK and WNT signaling pathways that are critical for normal neuronal homeostasis. These signaling pathway transcripts were also regulated by *Mettl3*, the m^6^A methyltransferase. We validated m^6^A changes using *Mettl3* knockdown and cross compared to other *Drosophila* studies; future non-antibody detection techniques would be important to apply.

Although m^6^A methylation primarily occurs in the 5’UTR of transcripts in *Drosophila*, *dTrmt10A* upregulation surprisingly skewed towards selective 3’UTR m^6^A regulation. Thus far, little is understood regarding why m^6^A is highly enriched in the 5’UTR of *Drosophila* transcripts vs 3’UTR as in mammals; however, *dTrmt10A* has uncovered that m^6^A regulation occurs in the 3’UTR of transcripts in *Drosophila*. Many critical signaling and neurogenesis transcripts such as *Sh, bru3, para,* and *pAbp* contain *dTrmt10A*-dependent m^6^A in the 3’UTR. Additionally, these transcripts were upregulated in brains with increased *dTrmt10A* expression, indicating they become more stabilized by loss of m^6^A modification. Transcripts of *Myc* and *Nrg* with 3’UTR m^6^A showed increased protein levels with *dTrmt10A* upregulation. These findings suggest that both 3’UTR and 5’UTR m^6^A serve functionally to keep transcript and protein levels low in the *Drosophila* brain. Furthermore, m^6^A marked transcripts are enriched in neural and dynamic signaling processes, indicating that one mechanism by which the brain regulates precise dynamics of neural signaling pathways may be through marking key transcripts with m^6^A for tighter control. Dysfunction in m^6^A is therefore predicted to have a larger impact on those transcripts critical for neural function. Additionally, we find that many 3'UTR m^6^A transcripts also have extended 3’UTR lengths in the *Drosophila* nervous system. A detailed analysis of *dTrmt10A* effect on alternative polyadenylation may be of interest, particularly within the context of the *Drosophila* nervous system, given the enrichment of the transcripts in learning, memory, and cognition.

We speculated that *dTrmt10A* may interact with a demethylase, similar to the mammalian enzyme. By mass spectrometry analysis of dTrmt10A IPed from head lysates and defining the interacting proteins, an obvious candidate was not revealed. The absence of a demethylase homologous to FTO in the fly suggests alternative mechanisms may be at play, such as interactions with RNA-binding proteins involved in decay. Additionally, dTrmt10A could impact proteins within the methyltransferase complex, which themselves have a more direct role on m^6^A modification; for example, dTrmt10A modulated fl(2)d levels. The mass spectrometry analysis showed dTrmt10A associates with key RNA degradation components including DCP1, Twin, and Rga. Thus, through interaction with dTrmt10A, these proteins may confer specificity and degradation impact of dTrmt10A on m^6^A targets. Furthermore, disease-associated protein Atx2, mutated in spinocerebellar ataxia 2, was found to co-IP with dTrmt10A. Atx2 is involved in RNA metabolism including RNA stability, RNA localization to stress granules and P-bodies, and interacts with other disease-associated RNA binding proteins such as TDP-43^59^. We also compared *dTrmt10A*-m^6^A modified transcripts to human FTO CLIP RNA targets. There was a significant overlap, and the degree of overlap was comparable to that of mammalian cells^27^. The overlapped targets are enriched for signaling pathways, similar to GO term analysis of all m^6^A targets^46^. Considering that *Drosophila* m^6^A is predominantly deposited in the 5’UTR, we were surprised to find more m^6^A transcripts modified in the 3’UTR that overlapped with FTO targets, indicating specificity in potential demethylase/degradation targeting.

### Cellular stress and m^6^A modifications

To reduce cellular burden during stress, global protein synthesis is repressed and non-essential RNAs are decayed or stored in stalled mRNA-protein granules. During this process, vital RNAs are translated for stress survival. In the recovery phase, stress mediated RNAs are in turn decayed and RNAs necessary for normal cell maintenance are once again transcribed and translated^36^. The process of stress response activation and resolution is a fine-tuned dance of RNA decay and translation, such that prolonged stress or failure to activate stress machinery is detrimental. RNA modifications have emerged as critical regulators of dynamic signaling in normal cell maintenance and also during stress recovery^20,31,38^. Particularly neurons, which are postmitotic and cannot remove harmful accumulations through cell division, show specialized mechanisms to combat cellular stress.

In mammalian cells m^6^A modification has been shown to increase upon heat stress in the 5’UTR of *Hsp70* to facilitate cap-independent translation^26^. Other studies in vitro indicate that *Hsp70* becomes co-transcriptionally marked by m^6^A, which leads to its degradation^39^. Furthermore during stress resolution, the lncRNA *Heat,* a transcriptional activator of HSF1, becomes heavily methylated by m^6^A, and Ythdc1 degrades the lncRNA to prevent stress gene activation^40^. In the *Drosophila* brain, *Hsp70* is m^6^A modified in the 5’UTR at baseline, and m^6^A is lost upon acute heat stress^46^. We hypothesize this limits Hsp70 translation at baseline and that the loss of m^6^A facilitates accumulation of the transcript for translation during stress. Thus, loss of m^6^A in the 5’UTR of *Hsp70* leads to elevated levels of Hsp70 protein, which may allow for a state of preconditioning of the brain to future stress. With *dTrmt10A* knockdown, we were surprised to see a dramatic increase in 5’UTR m^6^A on *Hsp70*—especially given that no other transcripts showed such a dramatic increase in m^6^A. These data indicate that stress resilience may be a particularly important target of *TRMT10A/dTrmt10A* gene mutation.

In summary, using m^6^A-IP sequencing, we find that *dTrmt10A* impacts 5’UTR m^6^A methylation on key stress chaperones, but *dTrmt10A* also highlights that 3’UTR m^6^A occurs and is regulated in *Drosophila*. These studies provide a framework for future insight into how m^6^A regulation is achieved at specific sites, like the 5’UTR vs 3’UTR. These data reveal that additional m^6^A regulators like *dTrmt10A* outside of the core complex components may profoundly modulate region specific methylation and/or demethylation. m^6^A regulation in the brain likely serves to fine tune signaling pathways for the continued maintenance of axons and dynamic synaptic connections. This comes at the cost of dampening the acute stress response, which compromises the overall ability of the brain to withstand insults. Given that mutations in *TRMT10A* are associated with developmental brain disease, some mutations that compromise *TRMT10A* function may also include impacts on m^6^A and stress resistance.

## Materials and methods

### *Drosophila* stocks and maintenance

A full list of *Drosophila* stocks used in this study are described in Supplementary Data 1. RNAi and UAS lines were generated by the Harvard Transgenic RNAi Project (TRiP) and the Vienna Drosophila Resource Center (VRDC)^60,61^, and stocks were obtained from the Bloomington *Drosophila* Stock Center, Indiana, USA. Crosses were performed at 25 °C and grown on standard cornmeal molasses agar. Driver lines used: *da*GAL4, *elav*Gal4, *nSyb*Gal4 as indicated per experiment. For all experiments male flies were used, for consistency in the experiments, and to avoid issues in food consistency due to egg laying of females.

### Brain dissections

Brain dissections were conducted as previously described^62^. Briefly, flies were anesthetized using CO_2_ and decapitated using forceps. The head was placed posterior side down and the proboscis was then removed using Dumont #5 fine forceps (Fine Science Tools, 11254-20). The brain was then gently popped out through the proboscis cavity, cleaned in PBS, and transferred to an RNAse free microfuge tube and PBS was aspirated. Brains were then ground in Laemmli Buffer (5 μL per brain, 10–20 brains for each sample) for Western immunoblotting, or in Trizol for RNA analysis.

### Western immunoblot analysis

Brain or head samples were homogenized in sample buffer of 1 × Laemmli sample Buffer (Bio-rad, 1610737), 50ul b-mercaptoethanol (Sigma, m6250), 1 × protease inhibitor (Roche, 11836170001), and 1 mM PMSF (Sigma, P7626). 5ul of sample buffer is added per brain, 7.5 ul added per head, and 40ul added per whole fly. Samples are boiled at 98 °C for 3 min, and then centrifuged at 1500 rpm for 3 min at room temp. Sample was loaded onto 15 well 1.0 mm 4%–12% Bis-Tris NuPAGE gels (Thermo Fisher, WG1401) with pre-stained protein ladder (Thermo Scientific, 22619). 1 brain, 1 head, or 8% of whole fly tissue is loaded on each lane per experiment. Gel electrophoresis was performed using Xcell Surelock Mini-Cell Electrophoresis System at 140 V, and transferred overnight onto a nitrocellulose membrane 0.45 μM (Bio-rad, 1,620,115), using a Bio-rad mini transblot cell at 90A for 16 h. Membranes were stained in Ponceau S (Sigma, P7170-1L), washed in DI water, and imaged with Amersham Imager 600. Ponceau S was washed off in 3 × 5 min in Tris-buffered saline with 0.1% Tween20 (TBST). Membrane was blocked in 5% non-fat dry milk (LabScientific, M08410) in TBST for 1 h, and incubated with primary antibodies with blocking buffer overnight at 4 °C. Following 3 × 5 min washed in TBST, membranes were incubated with HRP-conjugated secondary antibodies at 1:5,000 for 1 h at room temp in blocking solution. Membranes were washed 3 × 5 min in TBST and the signal was developed using ECL prime (Cytivia, #RPN2232) and detected using an Amersham Imager 600. Primary antibodies used: anti-tubulin (1:5000, DHSB, #AA4.3, Lot.5/31/18-44ug/ml), anti-Hsp70 (1:5000, Sigma, #7FB-SAB5200204-100uG, Lot.141002), anti-Mettl3 (1:5000, Proteintech, #15073-1-AP, Lot.Ag7110), anti-HSP40 (1:5000, Enzo Life Sciences, #ADI-SPA-400-D, Lot.04062141), anti-fl(2)d (1:10, #DSHB-9G2, Lot.10/18/18-42ug/ml), anti-dFmr1 (1:500, #DSHB-5A11), anti-Nrg (1:600, #DSHB-BP104), anti-Myc (1:50, #DSHB-P4C4-B10), anti-puromycin (1:1000, Kerafast, #EQ0001, Lot.200517). Rabbit anti-Ythdc1 (1:5000) was created by Vivitide against 18 residues of Ythdc1 (157–173 “CRTKIPSNANDSAGHKSD”)^46^. Secondary Antibodies used: Goat anti-mouse (1:5000, Jackson lmmunoResearch, #115-035-146, Lot.153978), Goat anti-Rabbit (1:5000, Jackson lmmunoResearch, #111-035-144, Lot.138306), Goat anti-rat (1:5000, Thermo Fisher Scientific, #A10549, Lot.2273679).

### RNA extraction

Tissue was homogenized in 200ul of Trizol (Thermo Scientific, #15596026) in RNase free 1.5 ml microfuge tubes (Thermo Scientific, #AM12400). 800ul of Trizol (Thermo Scientific, #15596026) was added to the tube and 200ul of chloroform (Fisher Scientific, #AC423555000) and was vigorously shaken for 20 s at room temp. Samples were left for 5 min at RT to form upper aqueous phase, and centrifuges at 4 °C for 15 min at 12,000×*g*. The Upper aqueous phase was transferred to a fresh RNase free tube. RNA samples were then processed using the Zymo RNA clean & concentrator -5 kit (Zymo, #R1013), using their RNA clean-up from aqueous phase after Trizol /chloroform extraction protocol plus on column DNase I treatment. RNA amount was measured using a nanodrop and integrity was validated through on an Agilent 2100 Bioanalyzer using an RNA nano chip.

### Real time PCR

400 ng RNA was used per cDNA reaction using the High Capacity cDNA Reverse Transcription Kit (Applied Biosystems, Thermo Scientific, #4368814). cDNA was then used for qPCR reactions set up with SYBR Green Fast Reagents, using a 384-well plates on the Applied Biosystems ViiA7 machine. Primers used are in Supplementary Data 1. Mean fold change was determined using the ∆∆Ct method. Each experiment used technical triplicates as well as three biological replicates, Graphpad prism 8/9 software was used for statistics.

### Stress sensitivity assay

Fly crosses were carried out at 25 °C. Adult flies were collected and aged to 6d post eclosion. Flies were anesthetized and transferred to clear plastic 13 ml vials, and cotton was placed at the 4 ml mark on the vials to concentrate the flies near the bottom. Each vial contained 20 flies. Flies were allowed to recover for 30 min, and then transferred to a water bath for mild non-lethal heat stress (30 min at 38.5 °C) or a longer heat shock (1–1.5 h at 38.5 °C) for a severe stressor to measure stress sensitivity. The flies were then transferred to normal food and allowed to recover overnight at 25 °C. After recovery, the percent of flies alive versus dead was recorded per vial.

### LC–MS/MS analysis of m^6^A levels

PolyA + RNA was extracted from brains, heads, and whole fly RNA using the NEBNext Poly(A) mRNA Magnetic Isolation Module (NEB, #E7490L). LC–MS/MS was conducted as previously described^27^. All quantifications were performed by using the standard curve obtained from pure nucleoside standards running with the same group of samples. Then, the percentage ratio of m^6^A to A or m^1^G to G was used to compare the different modification levels.

### m^6^A-IP sequencing

Total RNA was extracted from 200 *Drosophila* heads per replicate using Trizol/ chloroform extraction. PolyA + mRNA was obtained using NEBNext Poly(A) mRNA Magnetic Isolation Module. PolyA + RNA was fragmented using the NEBNext Magnesium Fragmentation Module (NEB, #E6150S) for 4 min at 95 °C for a 250 ng sample of polyA + RNA, and RNA was repurified using the Zymo RNA clean & concentrator -5 kit (Zymo, #R1013). 10% of the fragmented polyA + RNA was saved as an input control for sequencing. M^6^A -immunoprecipitation was done using the EpiMark N6-Methyladenosine Enrichment kit protocol with some minor alterations described. 30ul of protein G magnetic beads (NEB, #S1430) were washed and resuspended in IP buffer (150 mM NaCl, 10 mM Tris–HCL, 0.1% NP-40). 4ul of Synaptic Systems antibody (Synaptic Systems, #202003, Lot.2-119) was conjugated to protein G-magnetic beads (NEB, #E1611A, Lot.10015190) for 2 h at 4 °C. Beads/ antibody were washed twice in IP buffer. ~ 1 μg polyA + RNA was incubated with beads/antibody in IP buffer supplemented with 0.1% SUPERase-In RNase Inhibitor (Thermo Scientific; #AM2696) for 2 h at 4 °C. After incubation, RNA/beads/antibody are washed twice in IP buffer, twice in low salt IP buffer (50 mM NaCl, 10 mM Tris–HCL, 0.1% NP-40), and twice in high salt IP buffer (500 mM NaCl, 10 mM Tris–HCL, 0.1% NP-40). RNA is eluted from beads with 25 μl of RLT buffer twice and elution was pooled and concentrated using Zymo RNA clean and concentrator -5 kit (Zymo, #R1013). Libraries were made using SMARTer Stranded Total RNA-Seq Kit V2 without rRNA depletion (Takarabio, #634411) for IPed and input RNA, and sequenced using illumina HiSeq X series with 40M paired end reads (2 × 150 bp). Library preparation and sequencing was done by Admera Health. Three biological replicates per genotype and condition were done with NEB m^6^A antibody, and two biological replicates were done with Synaptic Systems m^6^A antibody.

### m^6^A enrichment analysis

Regions of m^6^A enrichment were found for each condition using MetPeak (v.1.1)^63^ with default parameters, using the input and m^6^A pulldown bam files as input, and with the FlyBase FB2019_05 annotation provided. Peak locations (5’ UTR, CDS, or 3′ UTR) were defined from the regions indicated by MetPeak as having significant m^6^A enrichment. If a peak was not contained in one region (i.e., if the peak is partly in the CDS and partly in the 3’ UTR), it was assigned to the region where more of the peak resided.

### Differential m^6^A peak analysis

Regions of differential methylation between two conditions (frequently called “*dTrmt10A*-dependent” or “m^6^A genes”) were found using RADAR (v.0.2.4)^64^ with input and m^6^A pulldown bam files as input, as well as the FlyBase FB2019_05 annotation. All replicates were used for differential peak calling. The minimum cutoff for bin filtering was 15, the cutoff was set as 0.05, and the Beta_cutoff was set as 0.5. Any region with an adjusted *p*-value < 0.05 was retained, and regions with a fold-change < − 1 from the control (KK Control) to the upregulation (UAS-*dTrmt10A*) at basal or heat shock (30 min) conditions were kept as *dTrmt10A*-dependent peaks. All other genes expressed in the brain that did not have *dTrmt10A*-dependent m^6^A were considered as “all other genes.” Full RADAR differential peaks with no cut-off are listed in Supplementary Data 2.

### m^6^A metaplots and genome browser visualization

Heatmaps and metagene plots showing the location of m^6^A enrichment on a specific set of genes were constructed with using pheatmaps (v.1.0.12) and meRIPtools (v.0.2.1). Specifically, the exons of all transcripts in each gene were collapsed using the GenomicRanges (v.1.44.0) function reduce^65^. Genes with a 5′ UTR or 3′ UTR shorter than 30 base pairs, a CDS shorter than 100 bp, or lacking a 5′/3′ UTR (i.e., lncRNAs) were not considered in this analysis. For each gene, the 5’ UTR and 3′ UTR were tiled in 30 evenly spaced bins, and the CDS was tiled in 100 evenly spaced bins. The number of input and m^6^A reads overlapping each bin was calculated and this number was divided by the bin width and library size and a normalization factor of one million to produce a normalized reads per million in each bin. For genome browser snapshots, separated input and m^6^A-IP tracks vere visualized. Tracks were made by first converting bam files to bigWig files using deepTools (v.3.5.1)^66^ bamCoverage using CPM normalization, then deepTools bigwigCompare with operation log2.

### GO and pathway analysis

GO analysis for genes with *dTrmt10A*-dependent m^6^A was conducted using FlyMine (v.53)^67^. The test correction was set to Holm-Bonferroni with a max *p*-value of 0.05. KEGG^49,50^ pathway analysis was done using the “enrichKEGG” function from ClusterProfiler (v.4.0.5) package in R^68^. A list of all genes with detectable expression was used as background for both GO and pathway analysis.

### RNA-seq analysis

Raw paired-end fastqs were processed with TrimGalore (v.0.6.6) (https://github.com/FelixKrueger/TrimGalore) with default settings to remove Illumina adapters and mapped using STAR 2.7.3a^69^ to the *Drosophila melanogaster* genome annotation dm6. Unmapped and improperly paired reads were filtered out of aligned bam files. Reads per gene in the FlyBase release 2019_05 were computed using an R script using GenomicRanges (v.1.44.0)^70^ function summarizeOverlaps that counts the number of reads overlapping with the exons of each gene in the default “union” mode. Differential expression analysis was performed using DESeq2 (v.1.32.0)^71^, with count files produced by summarizeOverlaps as input. PCA plots were made using the plotPCA function in DESeq2, with variance stabilized counts as the input. MA plots were constructed from the adjusted *p*-values and baseMean values output from DESeq2, and volcano plots were constructed from adjusted p-values and fold changes reported by DESeq2. Normalized counts produced by DESeq2 were used to show expression levels. Differentially expressed genes were considered to be any gene with a *p*-adjusted values of < 0.05.

### Actinomycin assay

Brains from KK Control, UAS-*dTrmt10A*, and *Trmt10A* RNAi adults were dissected and incubated in 250ul of Schneider's *Drosophila* Medium that was pre-warmed to 25 °C in RNase free 1.5 ml microfuge tubes. 12–15 brains were dissected for each timepoint. 169.5ug/ml actinomycin D (Sigma-Aldrich, A1410) was added to each sample. Samples were incubated at 25 °C shaking at 300 rpm for 4 h, and mixed by pipetting every 1 h. After the incubation time samples were collected, spun at room temp, washed in cold RNAse free PBS, and processed in Trizol. Brain total RNA was collected using trizol/chloroform and Zymo RNA clean and concentrator kit-5 (R1015). RNA was then used for RT-qPCR. 400 ng of RNA is used per cDNA reaction. cDNA is also quantified by Qubit ssDNA Assay (Invitrogen, Q10212) before use in RT-qPCR experiments.

### Puromycin assay

Food was made by mixing 600 µM puromycin (Sigma-Aldrich, P8833-100MG) with 2% agar + 5% sucrose. Adult flies were placed onto puromycin food for 24 h. Brains were dissected from flies after 24 h of feeding and samples were processed in for western blot analysis. Protein loading was determined by Ponceau S stain of membrane.

### Quantification and statistical analysis

Statistical tests used were performed on GraphPad Prism (v.9), and are indicated in the figure legend. *p*-values of < 0.05 were considered significant. Unpaired two-tailed t-tests were used when comparing 2 groups; One-way ANOVA was used when comparing multiple groups followed by Tukey’s post-test when each group was compared against every other group, Sidak’s post-test when pre-defined groups were compared to each other, or Dunnett’s test when comparing to a defined control sample. Two-way ANOVA was used when there were 2 factors in the analysis. One-sided Hypergeometric test was used to compare Venn diagram overlaps.

### Salivary gland immunohistochemistry

Third instar larva were dissected in PBST (0.05% Tween 20) and salivary glands were fixed in 2% paraformaldehyde (Electron Microscopy Sciences, #15713) in 45% acetic acid (Alfa Aesar, #36289) for 1 min at room temperature. Fixed salivary glands were loaded in 45% acetic acid on the slide glass and covered with siliconizing reagent (Sigmacote, Sigma-Aldrich, #SL2) coated coverslips to squash polytene chromosomes. Slides were snap frozen with liquid nitrogen and coverslips removed. Samples were blocked with 3% BSA (Sigma, #A7906) solution in PBST for 1 h at room temperature. Primary antibody against HA (Santa cruz, sc-805) and goat anti-rabbit IgG Alexa Fluor488 (Invitrogen, #A11008) were diluted with blocking solution in 1:50 and 1:200 ratio, respectively. VECTASHIELD mounting medium with DAPI (Vector Laboratories, #H-1500) was added at the last step to stain nucleic acids and mount samples. Imaging was conducted on a Leica STELLARIS 5 confocal microscope.

### ChIP-WB and RNase treatment

Fly brains (30 brains/sample) were dissected in cold PBS buffer and fixed in 1% formaldehyde (Fisher Scientific, #BP531) for 10 min at room temperature, and washed 3 times with PBS buffer. Fixed brain samples were homogenized in lysis buffer 1(50 mM HEPES pH7.5, 140 mM NaCl, 1 mM EDTA, 10% Glycerol, 0.5% NP-40, 0.25% Triton X-100), and treated with lysis buffer 2 (10 mM Tris pH8, 200 mM NaCl, 1 mM EDTA, 0.5 mM EGTA), then chromatin samples were collected after sonication in lysis buffer 3 (10 mM Tris pH 8, 100 mM NaCl, 1 mM EDTA, 0.5 mM EGTA, 0.1% Sodium Deoxycholate, 0.5% N-lauroylsarcosine). 10% of chromatin sample was used for input samples. Anti-HA (Santa Cruz, #sc-7392X, 2ug) and normal mouse IgG (Santa cruz, #sc-2025, 2ug) antibodies were added to Dynabeads Protein A (Invitrogen, #10002D) and incubated for 6 h at 4 °C. Then applied beads to chromatin samples for overnight incubation at 4 °C. Beads were washed and then denatured with LDS sample buffer (Invitrogen, #NP0007) at 95 °C for 5 min. Beads and supernatant were separated on a magnet stands, and the supernatant was used to perform western blot. For RNase treatment, RNase A/T1 mix (Thermo Scientific, #EN0551) was added to lysis buffer 2, and incubated with brain pellets for 2 h at 4 °C.

### Immunoprecipitation with dTrmt10A

To immunoprecipitate dTrmt10A from adult fly heads the Pierce Co-Immunoprecipitation kit (Thermo Scientific, #26149) was utilized according to manufacturer’s instructions. 15ug either anti-HA antibody (mouse, Santa Cruz, #sc-7392X) or mouse IgG (mouse, Santa Cruz, #sc-2025) was immobilized to agarose resin according to kit instructions. Heads of adult male flies expressing UAS-*dTrmt10A*-FLAG-HA with *da*Gal4 were isolated and homogenized for 1 min on ice in 200 uL of IP Lysis/wash buffer (Thermo Scientific) that was supplemented with cOmplete Mini EDTA-free protease inhibitor (Roche, #11836170001). 20 uL of sample was removed for input. Remaining sample lysate was pre-cleared with agarose resin (1 h at 4 °C with gentle rocking). Precleared lysate was incubated with antibody-coupled resin overnight (~ 18 h) at 4 °C with gentle rocking. Agarose resin was then washed three times with IP Lysis/wash buffer supplemented with protease inhibitor (10 min each with rotation at 1000×*g*, 4 °C). IP samples were eluted in either 50 uL for immunoblotting or 30 uL for mass spec analysis. Samples for immunoblotting were prepared in 4 × LDS Sample Buffer (Invitrogen, #NP0007), heat denatured at 90 °C for 6 min and electrophoresed on a 4–12% Bis–Tris gel and transferred to nitrocellulose. dTrmt10A signal was detected using anti-HA-peroxidase antibody (1:500 rat, Roche, #12013819001).

### Mass spectrometry analysis of dTrmt10A protein

dTrmt10A was immunoprecipitated from adult fly heads (80 per replicate, n = 3 biological replicates per antibody) as described above using anti-HA antibody or mouse IgG as control. Samples were run on NuPAGE™ 10%, Bis–Tris gel for 0.5 cm, and stained using Imperial™ Protein Stain (ThermoFisher, #24615). Mass spectrometry analysis was done by Wistar Proteomics and Metabolomics Facility. The entire stained gel regions were excised, reduced with TCEP, alkylated with iodoacetamide, and digested with trypsin, and tryptic digests were analyzed using a standard 1.5-h LC gradient on the Thermo Q Exactive Plus mass spectrometer. MS data were searched with full tryptic specificity against the UniProt *Drosophila melanogaster* proteome database (7/21/2022) plus the sequence provided (dTrmt10A-HA), and a common contaminant database using MaxQuant 1.6.3.3. Fold change was calculated based on intensity of HA signal to IgG signal, and intensity is the sum of the peptide MS peak intensities for the protein. To calculate the fold change, protein intensity values were processed by: Log2 transformation of the intensity values and replacing missing values (proteins not identified) with the minimum value of the dataset, for comparison between two sample groups, Log2 ratio, Fold change and Student's t-test *p*-value were calculated using the Log2 imputed intensity, the more stringent *q*-value (t-test *p*-value adjusted to account for multiple testing using Benjamini–Hochberg FDR) was also calculated. "High" confident identification of proteins with significant change ("Confident" column) refers to proteins satisfying the 4 criteria: minimum absolute fold change of 2, and *q*-value < 0.1, and identified by a minimum of 2 razor + unique peptides in HA-IP group, and detected in at least 2 of the HA-IP replicates. "Medium" confident protein identifications refer to proteins satisfying the 3 criteria: Identified by a minimum of 2 razor + unique peptides in HA-IP group, and detected in at least 2 of the HA-IP replicates, and not detected in any of the IgG-IP replicates.

### Supplementary Information

Supplementary Figures.

Dataset S1.

Dataset S2.

Dataset S3.

Dataset S4.

## Data Availability

The Raw sequencing data generated in this study have been deposited in the Gene Expression Omnibus GSE229430. Any additional inquiries can be directed to the corresponding author.
