# Supplementary material for: dTrmt10A impacts Hsp70 chaperone m6A levels and the stress response in the Drosophila brain
Source: Sci Rep. 2023 Dec 28;13:22999. doi: 10.1038/s41598-023-50272-4 (PMC10754819; doi:10.1038/s41598-023-50272-4)
Supplement: Supplementary file 1 — Supplementary Figures. [file 41598_2023_50272_MOESM1_ESM.pdf]

Supplementary Materials for

dTrmt10A impacts Hsp70 chaperone m^6A levels and the stress response in the *Drosophila* brain

Alexandra E. Perlegos[1], Xiuming Quan[2], Kirby M. Donnelly[2], Hui Shen[3], Emily J. Shields[4,5], Heidi Elashal[6,7], Kathy Fange Liu[6,7], Nancy M. Bonini[1,2,8*]

[1]Neuroscience Graduate Group, University of Pennsylvania, Philadelphia, PA, 19104, USA.
[2]Department of Biology, University of Pennsylvania, Philadelphia, PA, 19104, USA.
[3]School of Life Science and Technology, China Pharmaceutical University, Nanjing, Jiangsu, 210009, China.
[4]Epigenetics Institute and [5]Department of Cell and Developmental Biology, University of Pennsylvania Perelman School of Medicine, Philadelphia, PA, USA
[6]Department of Biochemistry and Biophysics, Perelman School of Medicine, University of Pennsylvania, Philadelphia, PA, 19104, USA.
[7]Graduate Group in Biochemistry and Molecular Biophysics, Perelman School of Medicine, University of Pennsylvania, Philadelphia, PA, 19104, USA.

This PDF includes:

Supplementary figures 1-5

Other supporting materials include the following:

Supplementary Data Files:
Supplementary Data 1 – *Drosophila* Lines and Primers
Supplementary Data 2 – *dTrmt10A* m^6A RADAR files
Supplementary Data 3 – GO terms and KEGG Pathway
Supplementary Data 4 – Mass Spec Analysis

Supplementary Figures

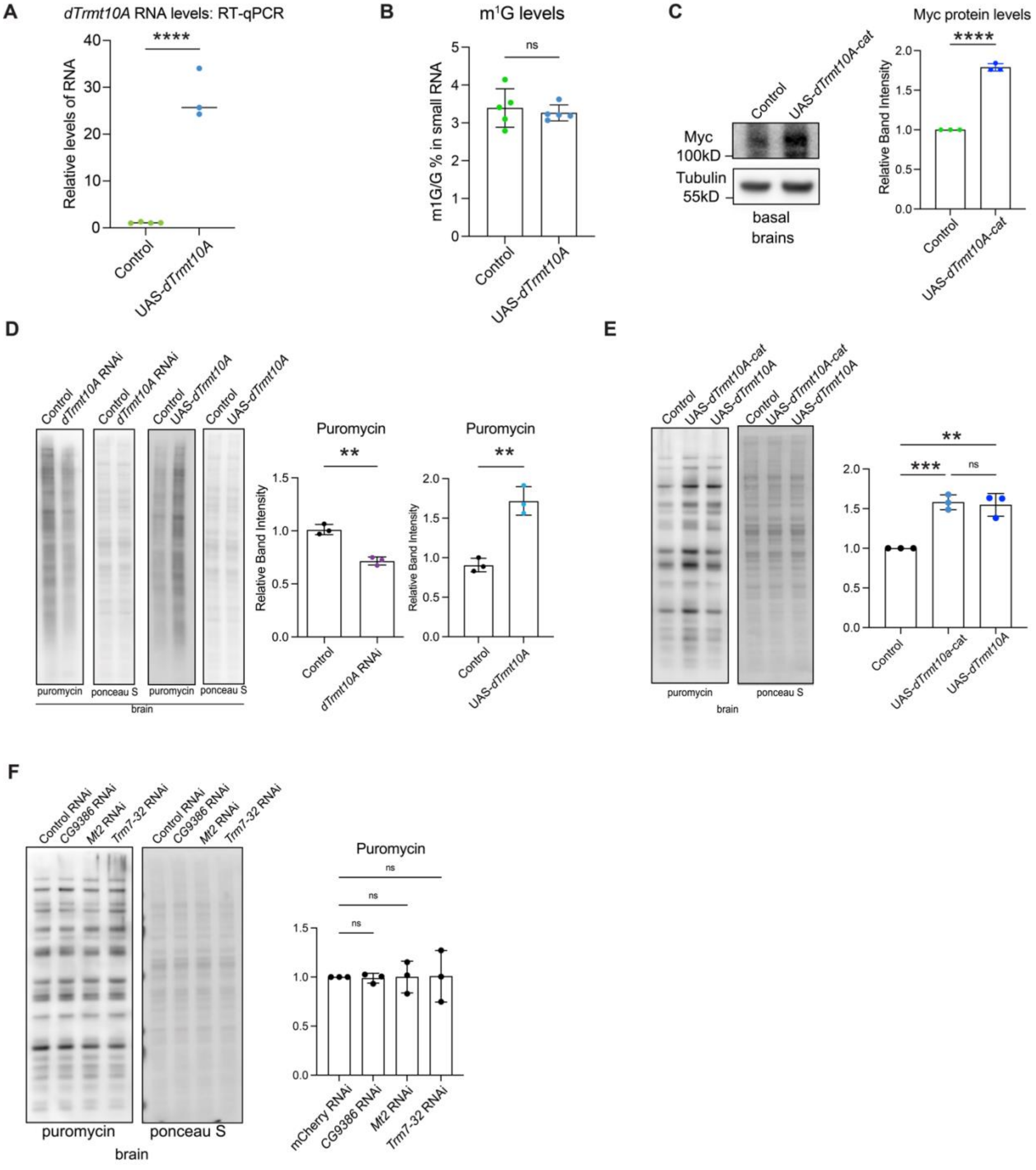

Supplementary Figure 1

(A) RNA levels of *dTrmt10A* were assessed by RT-qPCR to determine upregulation efficacy. *da*Gal4>KK control; *da*Gal4>UAS-*dTrmt10A*; n=3 biological replicates, 15 heads per replicate. Data are presented as mean, ****p<0.0001. (B) LC-MS/MS analysis of m^1G/G% levels from head small RNA in basal conditions, in *da*Gal4> KK control vs UAS-*dTrmt10A*, n=5 biological replicates, 30 heads per replicate. Data are presented as mean, t-test, ns=not significant. (C) UAS-*dTrmt10A-cat* increased protein levels of Myc in basal brain conditions. *da*Gal4>KK control vs *da*Gal4>UAS-*dTrmt10A-cat*, t-test, ****p<0.0001. (D) Puromycin assay of *da*Gal4> KK control vs *dTrmt10A* RNAi or Control vs UAS-*dTrmt10A*. Ponceau S staining shows total protein levels. n=3 biological replicates, 15 brains per replicate. Data are presented as mean ± SD, **p<0.01, t-test. (E) Puromycin assay of *da*Gal4> KK control vs UAS-*dTrmt10A-cat* mutant vs UAS-*dTrmt10A* brains. n=3 biological replicates, ***p<0.01, **p<0.01, One-way ANOVA. (F) Puromycin assay of *da*Gal4> mCherry RNAi control vs *da*Gal4>*CG9386* RNAi, *da*Gal4>*Mt2* RNAi, *da*Gal4>*Trm7-32* RNAi, One-way ANOVA, ns= not significant. n=3 biological replicates.

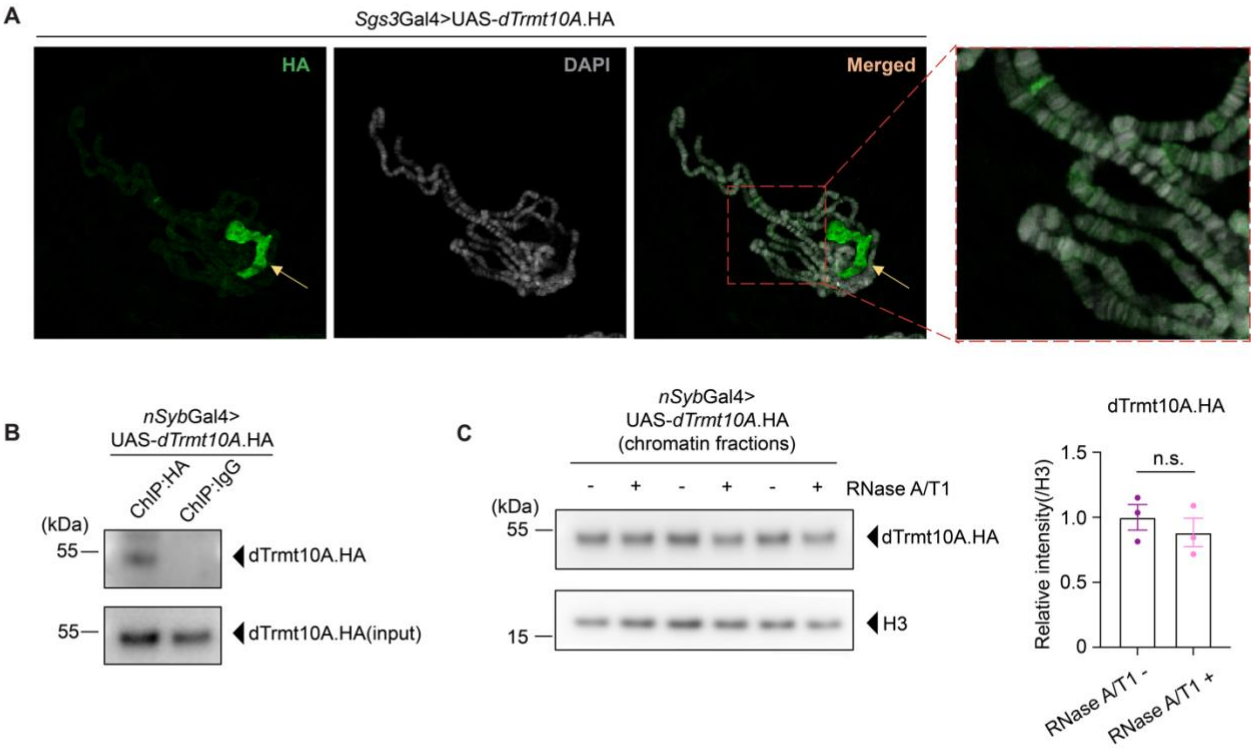

Supplementary Figure 2
dTrmt10A binds to chromatin. (A) dTrmt10A was expressed in *Drosophila* salivary glands (*Sgs3*Gal4 >UAS-*dTrmt10A*-FLAG-HA), and polytene chromosome preparations were immunostained for HA and DAPI. *dTrmt10A* is observed in the nucleolus (gold arrow) and bound to chromatin bands (red rectangle). (B) dTrmt10A.HA is present in the chromatin fractions (input) and immunoprecipitated from chromatin by HA antibody (ChIP). IgG is a negative control. (C) RNase A/T1 treatment has no effect on the level of dTrmt10A associated with chromatin, in the chromatin fractions by western immunoblot. N=3 biological replicates. Data are presented as mean ± SD, n.s. mean $p > 0.05$, Student's two-tailed t-test.

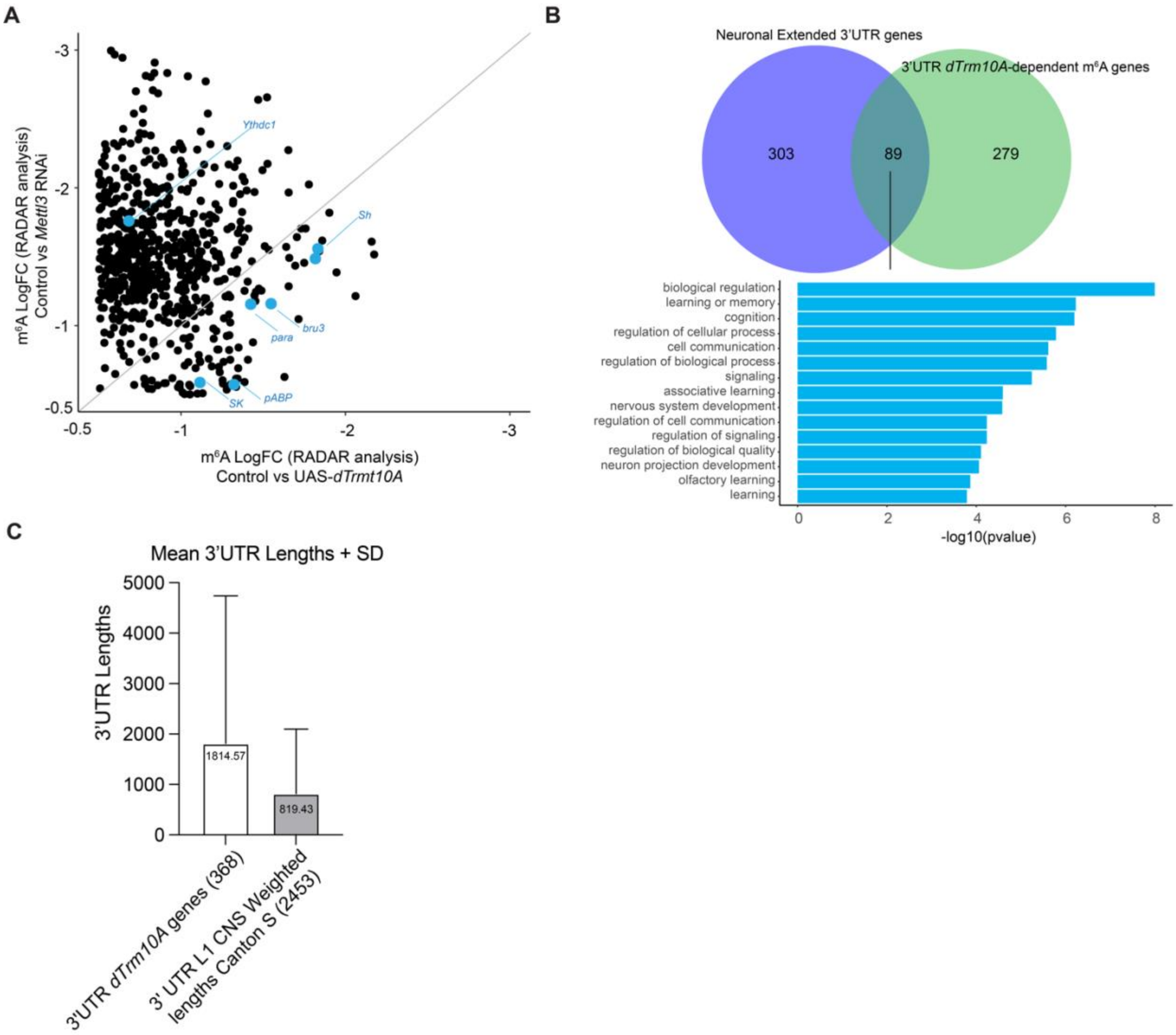

Supplementary Figure 3

(A) LogFC values of significant peaks determined by RADAR threshold analysis. Comparison of LogFC values upon *Mettl3* RNAi vs UAS-*dTrmt10A*. Blue dots refer to 3'UTR peak examples shown in Fig. 5E. (B) Comparison of extended 3'UTRs in neuronal cells[52] to 3'UTR *dTrmt10A*-dependent m^6A transcripts. 24% of T10A-dependent m^6A transcripts have extended 3'UTRs. Overlapped transcripts are enriched for learning and memory and cognition pathways. (B) Mean + SD 3'UTR lengths of the Trmt10A-dependent m^6A transcripts, compared to all 3'UTR weighted lengths in CantonS CNS [54].

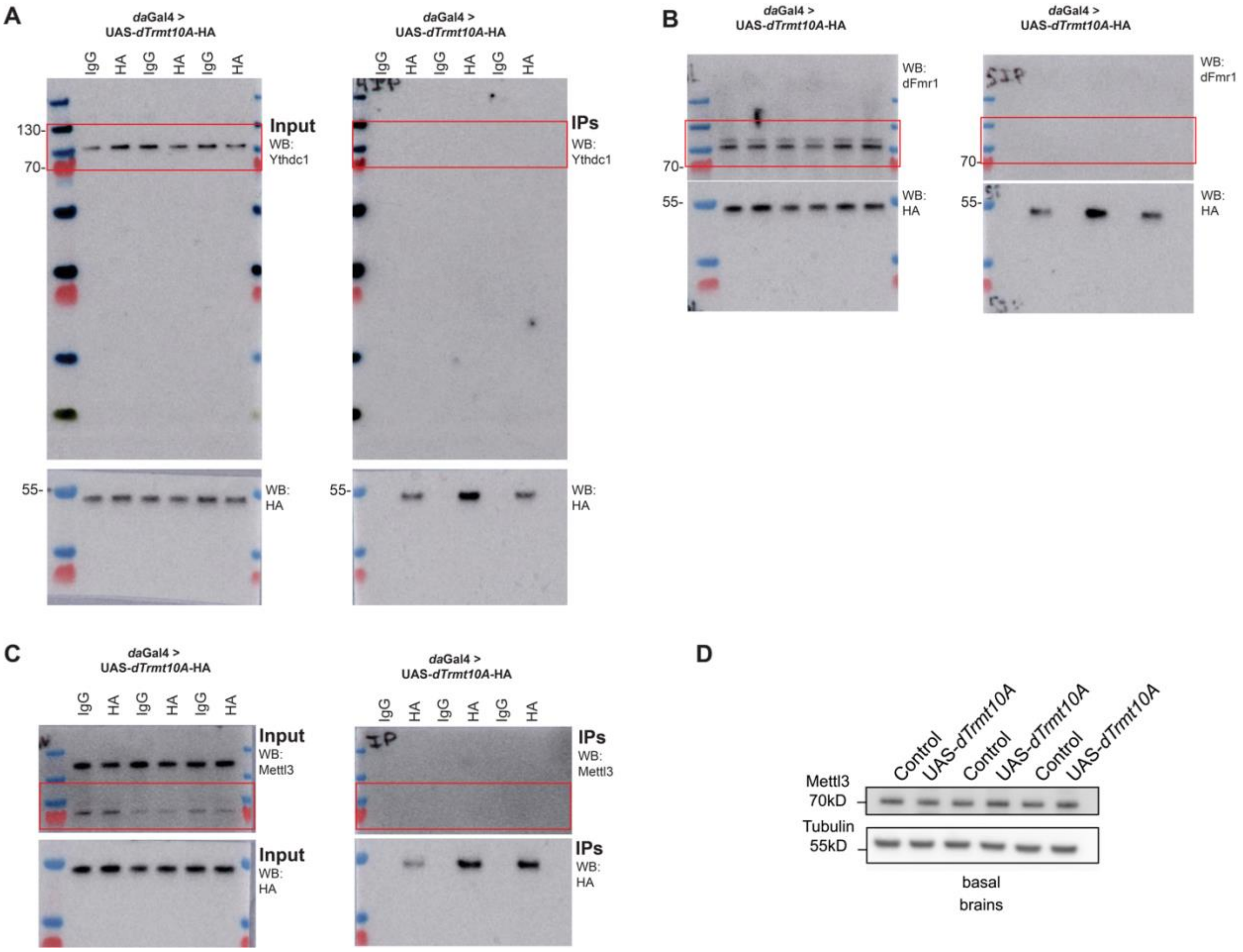

Supplementary Figure 4

(A) Immunoprecipitation of *dTrmt10A* (*da*Gal4>UAS-*dTRMT10A*-FLAG-HA) shows no IP interaction with Ythdc1. Left is input. Right is Immunoprecipitation comparing HA vs IgG control pulldown. Bottom is HA loading control for Input and IP. No interaction with (B) Fmr1, and no interaction with (C) Mettl3. (D) There is no change in the protein level of Mettl3 upon *dTrmt10A* upregulation (*da*Gal4>UAS-*dTrmt10A*), control (*da*GAL4>KK Control).

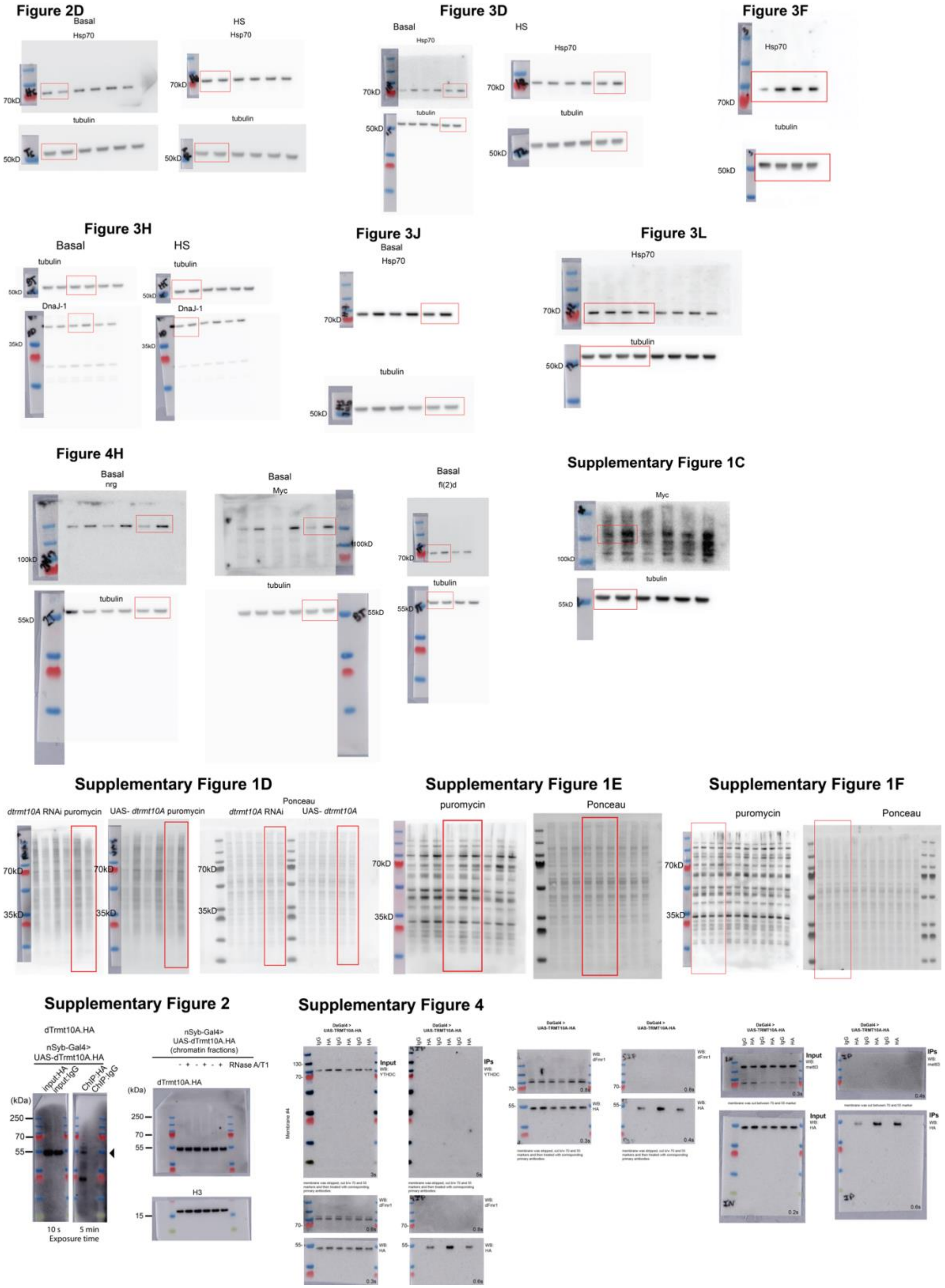

Supplementary Figure 5
Raw uncropped blots used in manuscript.
